# Analysis of *cycle* Gene Expression in *Aedes aegypti* Brains by *In Situ* Hybridization

**DOI:** 10.1371/journal.pone.0052559

**Published:** 2013-01-02

**Authors:** Samira Chahad-Ehlers, Carla Gentile, José Bento Pereira Lima, Alexandre Afranio Peixoto, Rafaela Vieira Bruno

**Affiliations:** 1 Laboratório de Biologia Molecular de Insetos, Instituto Oswaldo Cruz, FIOCRUZ, Rio de Janeiro, Brazil; 2 Laboratório de Fisiologia e Controle de Artrópodes Vetores, Instituto Oswaldo Cruz, FIOCRUZ, Rio de Janeiro, Brazil; 3 Laboratório de Entomologia, Instituto de Biologia do Exército, Rio de Janeiro, Brazil; 4 Instituto Nacional de Ciencia e Tecnologia em Entomologia Molecular, CNPq, Rio de Janeiro, Brazil; Karlsruhe Institute of Technology, Germany

## Abstract

Even though the blood-sucking mosquito *Aedes aegypti* is one of the most important disease vectors, relatively little is known about the molecular mechanisms underlying processes involved in the temporal pattern of its activity and host seeking behavior. In this study, we analyzed the expression of the *cycle (cyc)* gene, one of the core components of the circadian clock, in *Ae. aegypti* brains by *in situ* hybridization at two different time points in light-dark conditions and compared the results with those obtained using a quantitative PCR assay (qPCR). Within the brain, differential labeling was detected according to distinct areas empirically pre-defined. Six out of seven of these areas showed significantly higher staining at ZT3 (three hours after light-on) compared to ZT11 (one before light-off), which is consistent with the qPCR data. Predominant staining was observed in three of those areas which correspond to positions of the optical and antennal lobes, as well as the region where the neurons controlling activity rhythms are presumably localized.

## Introduction

The daily rhythms in behavior and metabolism of mosquitoes and other insects are controlled by endogenous circadian clocks [1], [2]. When isolated from temporal cues, organisms exhibit rhythms that persist under constant conditions, with a free-running period that is closed to 24-hours (circadian rhythms) and exhibit temperature-compensation, maintaining a similar period over a broad range of temperature. In addition, these rhythms are synchronized (entrained) to the environment by external stimuli such as light-dark cycles [2].

The *Drosophila melanogaster* central clock controlling activity behavior is composed by two main groups of neurons located dorsally (subdivided in dorsal neurons – DN1, DN2 and DN3 clusters) and laterally (subdivided in dorsal lateral neurons (LNd), ventral large lateral neurons (l-LNv), posterior lateral neurons (LPN) and ventral small lateral neurons (s-LNv) clusters), while in mammals the clock is located in the suprachiasmatic nucleus (SCN) of the hypothalamus [3], [4].

The current consensus model for the molecular mechanism underlying most circadian oscillations involves a number of clock genes, whose mRNA and protein products rely on negative autoregulatory feedback loops. In *D. melanogaster*, the clock is controlled by regulatory loops involving the core genes *period* (*per*), *timeless* (*tim*), *Clock* (*Clk*), *cycle* (*cyc*), *vrille* (*vri*), *Par domain protein* 1 (*Pdp1*) and *clockwork orange* (*cwo*) [5]. In the first and main loop (PER/TIM loop), transcription of *per* and *tim* is activated by the CLK-CYC heterodimer that rhythmically target E-box sequences within the *per* and *tim* promoters. After accumulating in the cytoplasm, PER and TIM are translocated to the nucleus, where PER or the complex PER-TIM will block the activity of CLK-CYC and the transcription of their own genes *per* and *tim* [5]–[7]. In the second feedback loop, *Clk* oscillating expression is generated through VRI (transcriptional repressor) and PDP1 (transcriptional activator) activities, while in the third loop the protein encoded by *cwo*, which is activated by CLK-CYC, suppresses its own expression and that of *per*, *tim*, *vri* and *Pdp1* [8]–[10]. It is worth noticing that, in *Drosophila*, *cyc* expression does not oscillate with any detectable amplitude at the RNA or protein level [11].

In mouse (*Mus musculus*) BMAL1, the mammalian orthologue of CYC, is the partner of mammalian CLOCK. Interestingly, *Drosophila* CYC is missing an activation domain called BCTR (“BMAL C-terminal region”) that is found not only in mammals but also in a number of other insects [12]–[16]. The interaction between CYC/BMAL1 and the mammalian-like protein CRY2, which act as a transcriptional repressor instead of PER in many insects as butterflies, is made through the BCTR domain [17], [18]. In addition, unlike *Drosophila* but similarly to Bmal1, *cyc* displays rhythmic daily expression in most insects [12], [19], [20], including the blood-feeding mosquito *Ae. aegypti* (Linnaeus, 1762), the main vector of Dengue and Yellow Fever [14].

In addition to its role in circadian regulation, *cyc* also integrates other *Drosophila* behaviors such as wake and sleep. Without the CYC protein, total amount of sleep decreases considerably [21]. It also induces sleep suppression during starvation and is involved in the complex decision of sleep or seek for food [22]. Particularly, the mushroom body is the main structure required for these behaviors [23] and is also engaged on the odor and visual memories [24].

In heads of female *Ae. aegypti cyc* shows a distinctive daily rhythm pattern of mRNA fluctuations in LD12∶12 (12 hours of light and 12 of dark) and DD (constant darkness) conditions, with a peak in the early morning (around ZT3 in LD) and a trough in the late day - early evening (around ZT11 in LD) [14]. However, no information on the spatial distribution of the clock neurons and *cyc* expression within the insect’s brain or the particular fluctuation of the gene expression in the putative brain areas where the “central” mosquito clock controlling activity rhythms probably resides [25] is yet available. In this paper we developed and applied an *in situ* hybridization method to visualize the expression pattern of *cyc* mRNA in the *Ae. aegypti* brain sites. The method and results described here provide a starting point for the characterization of specific clock gene signals in the mosquito brain.

## Materials and Methods

### Insects

*Ae. aegypti* were derived from a laboratory colony (Rockefeller strain) provided by *Laboratório de Fisiologia e Controle de Artrópodes Vetores* (LAFICAVE/IOC) & *Instituto de Biologia do Exército* (IBEx), Rio de Janeiro, Brazil. The mosquitoes used in the *in situ* hybridization and quantitative Real-time PCR (qPCR) experiments were maintained in an entrainment period of, at least, three days in a LD12∶12 cycle (12 hours of light followed by 12 hours of darkness). After entrainment, mosquitoes were collected at different time points (see below).

### Probe Synthesis

The cDNA template for *cyc* probes were amplified with 3aecyc1a (5′ GTCAGTTTGTTCAGCTTCC 3′) and 5aecyc1a (5′ TTCTCCCTGCAAGATCTACC 3′) primers in the cycling conditions as follows: 30 seconds at 95°C, 30 seconds at 55°C and 90 seconds at 72°C for 35 cycles. A 800 bp PCR product (correspondent to the region immediately after the transcription start site) was cloned in pGEM Easy Vector (Promega). The sense and antisense probes were transcribed using SP6/T7 Transcription kit (Roche), according to the manufacturer protocol.

### *In situ* Hybridization (ISH) and Image Analysis

*In situ* hybridization assays were done using biotinylated antisense RNA probes. We dissected brains collected at ZT3 and ZT11 (20 brains per time point) and fixed in Paraformaldehyde (PFA) 4% for 5 hours. After dissection, brains were washed with Phosphate Buffer (PB) with 0.5% Triton X-100 3 times for 15 minutes each and permeabilized with 1.25 µg/ml Proteinase K (GE Healthcare). Proteinase K reaction was stopped with 2 mg/ml glicine for 5 minutes and brains were washed with PB 1× with 0.1% Tween-20 (PBT) for 5 minutes. After washing, brains were again fixed with PFA 4% in PBT 1× for 20 minutes and washed in PBT 2 times for 10 minutes each. Subsequently, brains were incubated with PBT 1× at 60°C for 50 minutes and then with Hybridization Solution (50% Formamide, 5× SSC, 10 µg/ml Salmon Sperm DNA, 50 µg/ml Heparin and 1% Tween-20) at 60°C for 1 hour. The brains were then incubated with *cyc* antisense RNA biotinylated probe (100× diluted in Hybridization solution) at 60°C overnight (sense probe was used as control). In the following day, brains were washed with Hybridization solution twice for 15 minutes at 60°C. Then, brains were washed in a series of 3∶2, 1∶1 and 2∶3 of Hybridization solution: 2× SSCt (SSC +0.1% Tween-20) for 15 minutes each at 60°C. Later, brain were washed once with 2× SSCt for 15 minutes at 60°C and then with 0.2× SSCt for 15 minutes at 60°C. After that, brains were washed in a series of 3∶2, 1∶1 and 2∶3 of hybridization solution:0.2× SSCt for 15 minutes each at 60°C. Next we washed the brains with PBT 1× three times for 5 minutes each and blocked with 5% normal goat serum (NGS) in PBT1× at room temperature for 2 hours. After blocking, the brains were incubated with anti-biotin-POD antibody (Roche) 1000× diluted in PBT1× at 4°C overnight. In the next day, brains were washed with PBT1× for 30 minutes at room temperature and then revealed with 0.7 mg/ml 3–3′diaminobenzidine (DAB) (SIGMA) until color development (about 1 minute).

The mosquitoes used in the *in situ* hybridization assays were collected at ZT3 (*Zeitgeber* 3–3 hours after lights on) and ZT11 (*Zeitgeber* 11–11 hours after lights on). Around eight labeled brains per timepoint were captured in a stereomicroscope Discovery V.12 (Zeiss) and posteriorly analyzed though ImageJ software (NIH/USA). The pixels from different brain sections were quantitated by densitometry and data were then normalized to plot the relative expression of *cyc* gene in the two *Zeitgebers*. The differences seen between peak and trough labeling were tested by t-test using R software [26].

### RNA Extraction, cDNA Synthesis and Quantitative Real-Time PCR Assays

The mosquitoes used in the quantitative Real-Time PCR (qPCR) assays were collected every 2 hours over a 24-hour period starting at ZT1 (*Zeitgeber* 1–1 hour after lights on) and finishing at ZT23 (*Zeitgeber* 23-11 hours after lights off). Around 10 mosquitoes per time point were sampled. Total RNA from the heads was extracted using TRIzol reagent (Life Technologies) according to the manufacturer protocols and further purification with LiCl 7.5M [27]. The RNA samples were reversely transcribed into cDNA using TaqMan Reverse Transcription Reagents (Life Technologies). The reaction was performed with oligo-dT, and in the following temperature conditions: 25°C for 10 min, 48°C for 1 h and 30 min and 95°C for 5 min. qPCR assays were performed in ABI Prism 7700 (Applied Biosystems) using the SYBR Green PCR Master Mix (Life Technologies). Reactions were carried out for *cyc* and *rp49* genes, the latter used as the reference gene. Primers used for amplification of both genes and cycling conditions were previously described elsewhere [14]. Data analysis was carried out by the ΔΔCq method and Excel software and statistically tested by ANOVA using R software [26].

## Results

**Figure 1:**
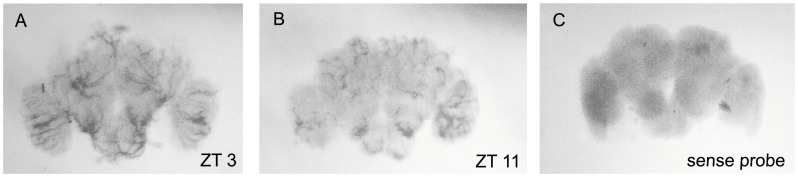
Whole mount *in situ* hybridization in *Ae. aegypti* male brains. Brains collected at ZT3 and ZT11 corresponding respectively to peak (A) and trough (B) *cyc* gene expression in LD12∶12 according to qPCR, were hybridized with a biotin-labeled RNA probe. It can be noted that in (A), where *cyc* expression is higher, that there are more labeled cells than in (B), where *cyc* expression is very low and nearly absent in some regions. These cells may correspond to some neuron clusters similar to the *Drosophila* oscillator cells. Sense probe (C) was used as control.

We performed whole mount *in situ* hybridization (ISH) experiments in female and male brains using a labeled RNA probe of the *cyc* gene. However, we found technical difficulties in working with brains of females due to cibarial dilator pump and the pharyngeal dilator pump. Both muscular structures, crucial for blood-feeding, are very attached to the brain and are dark brown colored, which difficult the dissection and staining [28]. Males don’t feed on blood and therefore have much less developed cibarial and pharyngeal dilator pumps, and for this reason had been proven much more suitable for our assay. Therefore we concentrated our analysis on male brains comparing the labeling in brains collected at ZT3 and ZT11. Around eight labeled brains per time point as well as sense probe negative controls were analyzed and the results are illustrated in Figure 1 which shows representative examples of each case. We observed in male brains collected at ZT3 many labeled areas, which includes neuron clusters in the optical lobe, the antennal lobe and in the protocerebrum. Conversely, fewer regions were labeled in male brains collected in ZT11. Added to this, the labeling was less intense in these regions. Sense probe was used as control and no labeling was observed, indicating the probe specificity.

**Figure 2:**
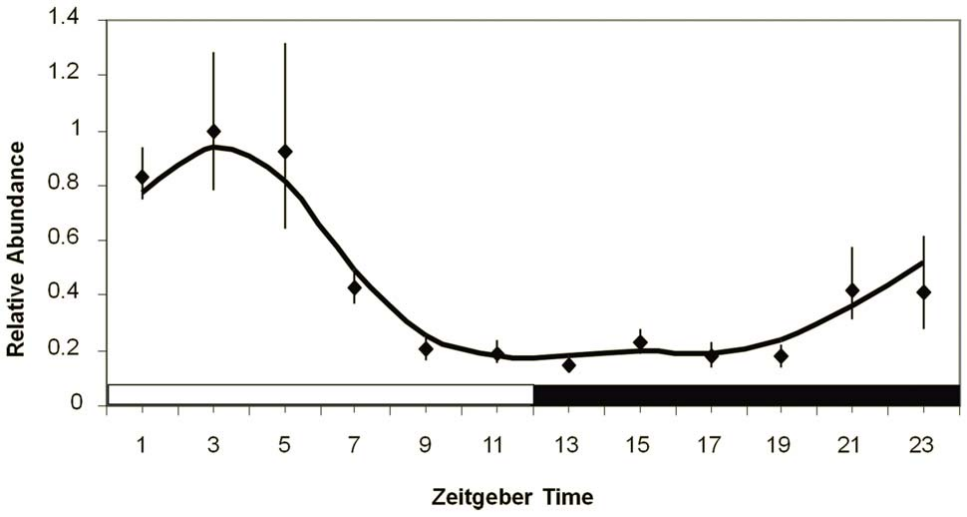
Circadian expression of *cyc* gene in *Ae. aegypti* males. The graph shows gene expression in a light:dark (LD12∶12) cycle, where the white horizontal bar represents 12 hours of light while the black bar represents 12 hours of dark. The y-axis indicates the relative mRNA abundance and the x-axis the time points. Bars below the x-axis indicate the light regime: white = lights on (day in LD cycles); black = lights off (night in LD cycles). These graphs were obtained through averaging data from 5 independent experiments. Lozanges depict the average value in each time point, and the curves were obtained through a 3-point weighted moving average of these values (weight of 2 for the central time point. Bars represent the standard error of the mean. ANOVA indicates significant difference between time points (F_11.60_ = 7,756; P<0.001).

The difference in signal intensity between ZT3 and ZT11 is consistent with our previously published results on *cyc* expression in *Ae. aegypti* females heads [14]. Nevertheless, we confirmed that a similar pattern is also seen in males. Figure 2 shows *cyc* gene expression in light-dark cycles detected through qPCR assays in *Ae. aegypti* males. Expression levels are high during the early light phase (with the peak expression in ZT 3) and decrease towards the end of the light phase reaching a low expression level in ZT11.

**Figure 3:**
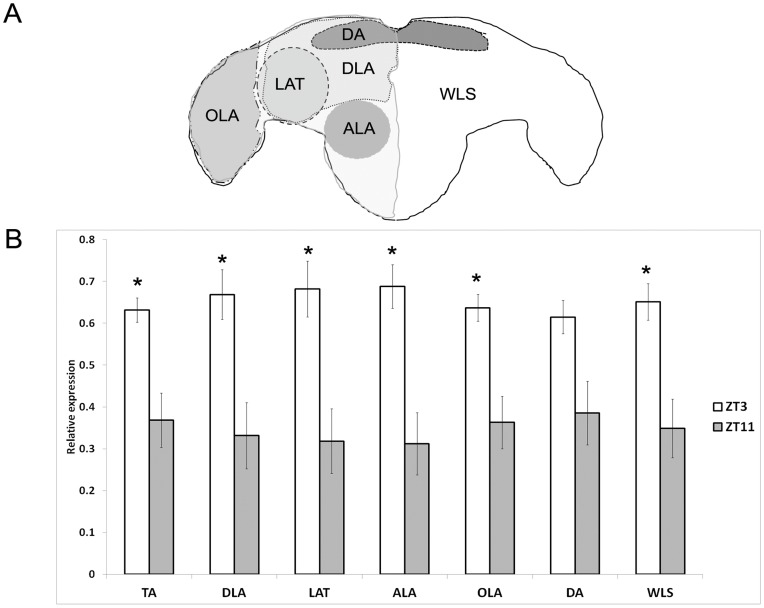
Schematic representation of *Ae. aegypti* brain (A) and relative *cycle* gene expression in brain regions (B). To compare the differences between peak (ZT3) and trough (ZT11) *cycle* expression in brains, we arbitrarily divided the brain in regions and analyzed them using ImageJ software. A t-test was used to compare the two time points for the different regions and all but the dorsal area presented significant differences (P<0.05), marked by asterisks. TA: total area, DLA: Dorsolateral area; LAT: Lateral area; ALA: Antennal lobe area; OLA: Optical lobe area; DA: Dorsal area; WLS: Whole Left side. Brain scheme was based on Ignell and coauthors work [44].

To evaluate in more detail the observed differences in ISH assays illustrated in Figure 1, we converted the labeling data into densitometry values using the ImageJ software and compared the distinct brain regions. Based on the current knowledge on the *Ae. aegypti* brain [29], we divided it into the following regions: total area (TA), Dorsolateral area (DLA), Lateral area (LAT), Antennal lobe area (ALA), Optical lobe area (OLA), Dorsal area (DA) and the Whole Left Side (WLS) (Figure 3A). Anatomically, DLA involves the superior protocerebrum, mushroom bodies, lateral accessory lobes and central complex. DLA were individualized into two other regions, LAT and DL. One includes lateral protocerebrum and the other comprises the superior medial protocerebrum with the top part of the central complex, pars intercerebralis and the calyx of mushroom bodies. The lateral regions of protocerebrum are connected to the optic lobes. This area, here called OLA consists of three neuropils, the lobula, medulla and lamina. The other individualized area is located in the deutocerebrum, another ganglion of the brain, where the antennal lobes (ALA) are located. The WLS includes the whole left side of the brain with the three cerebral ganglia: protocerebrum, deutocerebrum and tritocerebrum.

We thus compared the labeling intensity between each area in ZT3 and ZT11. All regions but the dorsal area (DA) showed statistical differences between peak (ZT3) and trough expression points (P<0.05), confirming the qPCR data (Figure 3B).

Finally, to confirm the specificity of signal intensity differences between ZTs with *cyc* we use a *per* gene probe. The ISH results show that *per* oscillates in antiphase to *cyc* transcript (Figure S1) and this is consistent with previous results obtained using qPCR to characterized mRNA circadian clock gene expression in *Ae aegypti* heads [14].

## Discussion

Circadian activity rhythms are controlled by the central clock that lies in the central nervous system. In insects, we know from the model Drosophila that this central clock is composed by genes (clock genes) whose transcripts and protein products fluctuate with daily (circadian) rhythms [5]. CYC together with CLK are the key components that form the positive factors of the main autoregulatory feedback loop. As a dimmer, they are responsible to activate promoters of a set of clock genes which directly or indirectly mediate output pathways for the control of behavior, physiology and development [5], [30]. The temporal profiling at the mRNA level in *Ae. aegypti* shows that *cyc* is rhythmically expressed in females [14] as well as in male heads (see results), both oscillating in anti-phase to that of *per* transcript [14]. The results found for *Clk* suggest this gene also cycles in *Ae. aegypti* albeit with a weaker rhythmicity [14], [31], similarly to what is observed in *Anopheles gambiae* microarray [32]. *cyc* robust cycling pattern in the heads of *Ae. aegypti* suggests an important role in the clockworks as for the mammalian Bmal1, which exert significant contribution in sustaining the circadian periodicity of the transcription of important clock genes [33].

The autofluorescence of *Ae. aegypti* tissues [34] have limited the application of standard fluorescence *in situ* hybridization techniques making it a major obstacle for the detection of fluorescent label probes in brains. For the same reason, we were unable to perform fluorescent immunocytochemistry. Actually, our preliminary works also revealed that our anti-*Drosophila* antibodies did not yield satisfactory results in immunocytochemistry assays in this mosquito brain (data not shown). Nevertheless, detectable levels of staining by ISH were observed in the present work by using biotinylated antisense RNA probes. Our results showed that the staining levels from *in situ* using antisense oligonucleotide for *cyc* corroborated the qPCR expression for peak and trough time points, also validated by the staining with sense control probe. As observed, intense hybridization signals were detected at ZT3 in most analyzed areas of the brain compared to ZT11. The specificity of signal intensity differences between ZTs with *cyc* was also supported by the analysis of a second different probe (*per*). Consistent with previous findings in *Ae. aegypti* heads [14], *per* oscillates in antiphase to *cyc* mRNA as shown by ISH (Figure S1). These data strongly suggest that the enhanced expression in *cyc* transcript is rather specific, even though the analysis has revealed lower signal intensity in some areas between ZT 3 and ZT11.

Whereas the results obtained for *cyc* expression using qPCR technique are attributed to the entire head, the analysis by *in situ* indicated possible regions of the brain responsible for this cyclical event. Particularly, one of the areas herein nominated as lateral area (LAT) showed this difference of staining. The area delimits the protocerebrum between the optic lobe (OLA region) and the central brain, and it is where LNd and LNv are presumably localized. In *Drosophila* these neurons are implicated as the major pacemaker cells being involved as neuronal substrates for the M and E oscillators, *i.e.*, for adaptation to different photoperiods [3], [35]. Actually, the principal circadian pacemaker controlling activity rhythms is attributed to s-LNvs cells because they are able to keep rhythmic locomotor behavior under constant darkness (DD) [36].

Other than LNs area, *cyc* mRNA is highly expressed in the optical lobe (OLA) and antennal lobe (ALA) with different intensity between ZT3 and ZT11. Considering the magnitude of staining observed in these two areas (OLA and ALA), this indicates that a great contribution for the cycling observed in the entire head by qPCR and by the differences at the larger areas analyzed by *in situ* (DLA and WLS) is perhaps attributed to the eye and the antennal base structures, besides the ventral lateral neurons at LAT area. In fact, differences in expression according to tissue or cell type are clearly observed, for example, in the mammalian SCN where clock oscillation is considered a property of some, but not all SCN cells [37]. The same holds true for the *Drosophila* model. Differential expression is restricting to specific neurons at LNv and LNd [3], [36]. Despite of this, total head extracts is widely used in qPCR analysis for targeting cycling clock RNAs, which assumes that a given expression profile of a clock gene from head may be reflecting the signal of clock neurons. Accordingly, qPCR of clock mRNAs from dissected clock neurons shows relatively similar phase to those from heads, but amplitude is lower in heads [38].

The Dorsal Area (DA) represents the probable location of the dorsal neurons (DNs). Like other areas analyzed here, the DA was strongly labeled. Although the observed difference between the two ZTs was not significant in DA, the overall trend was similar to the other areas of the brain.

The region of DLA between DA and ALA showed weak staining. This is particularly interesting because the gamma and beta lobes of the mushroom bodies are located in that area. However, since projections are observed from LA towards DA (see Figure 1), it seems a reasonable assumption, concerning to mushroom body, that *cyc* mRNA is more expressed in the calyx neuropil. This pattern of neuronal circuitry is consistent with that evinced in the cricket *Allonemobius allard* [39] and seems to be associated with a crucial role in olfactory learning in insects [24].

In this study we have made an attempt to identify which regions of the *Ae. aegypti* brain are responsible for reflecting *cyc* mRNA oscillation detected with qPCR in heads. The *in situ* method applied here probably has a limitation in resolution not allowing one to distinguish neurons individually. However, we do observed difference in expression within restricted brain areas. Of interest, therefore, is the staining extension observed in the primary olfactory neuropil, the antennal lobe. The signals were more concentrated at the periphery, not whole lobe, being unexpressed in the center and upper part. Previous report on mRNA expression of blue light sensor *cry* reveled a cluster of neurons in the lateral area of the antennal lobe in tephritid fruit fly [40], which may be a fraction of the neurons stained herein by *cyc* probe. In the same way, the optic lobe showed certain specific staining in the present study particularly at ZT3 (time point of more intense signal) with strongest expression at the central part crossing all over this structure. With this method of study it is too early to say whether this signal may underlie the connection between elements linked to visual stimuli and central clock in *Ae. aegypti*, although there are evidences that LNvs sends projections into the medulla neuropil of optic lobe in *D. melanogaster* [41], [42].

*Ae. aegypti* is the most important vector of dengue and yellow fever viruses and understanding physiological processes that involve host seeking behavior is an important step to prevent pathogen transmission. Considering that the genes controlling blood-feeding are under circadian control in mosquitoes [43], it is of interest to investigate the degree to which clock genes contribute to signaling within the clock neurons and their targets.

Our current and previous results [14] show that *cyc* transcription is expressed with a circadian fashion in *Ae. aegypti* heads, with no distinction of gender. Tissue specificity signals hidden by qPCR assay were at least partially unraveled by *in situ* hybridization showing heterogeneity in staining pattern within the brain. Six out of seven areas of interest empirically pre-defined showed significant higher staining by the *cyc* mRNA anti-sense probe at ZT3 compared to ZT11. Particularly, predominant staining was observed in three areas which correspond to positions of lateral neurons and optical and antennal lobes. Accordingly, the detection of differential expression of *cyc* mRNA in brain areas involving visual and olfactory detection of food source, such as optical and antennal lobes, indicates the need to investigate the role of clock genes on these complex structures.

## Supporting Information

Figure S1 **Whole mount in situ hybridization in** ***Ae. aegypti*** **male brains using a** ***period*** **RNA probe.** Brains collected at ZT3 and ZT17 corresponding respectively to trough (A) and peak (B) *per* gene expression in LD12∶12 according to previous qPCR data [14], were hybridized with a digoxigenin-labeled RNA probe. It is worth to note the labeling pattern seen with the *per* probe is in antiphase with the labeling pattern seen in Figure 1. Sense probe (C) was used as control. To compare the differences between peak (ZT17) and trough (ZT3) *period* expression in brains, we arbitrarily divided the brain in regions (left) and analyzed them using ImageJ software. A t-test was used to compare the two time points for the different regions and some regions presented significant differences between the two time points, as the Antennal lobe area (P<0.05) and the Dorsal area (P<0.01), marked by asterisks (right). The other regions presented a borderline statistical difference. TA: total area, DLA: Dorsolateral area; LAT: Lateral area; ALA: Antennal lobe area; OLA: Optical lobe area; DA: Dorsal area; WLS: Whole Left side. Brain scheme was based on Ignell and coauthors work [44]. The *period* probe synthesis was done in the same way as the *cycle* one (See Materials and Methods), but using the following primers: Forward –5′ GAGCTCCATATTTTGAGACATC 3′ and Reverse: 5′ TATGAAAGACCGTCCAAGC 3′.
(TIF)
