# Supplementary figures and images for: Analysis of cycle Gene Expression in Aedes aegypti Brains by In Situ Hybridization
Source: PLoS One. 2013 Jan 2;8(1):e52559. doi: 10.1371/journal.pone.0052559 (PMC3534671; doi:10.1371/journal.pone.0052559)

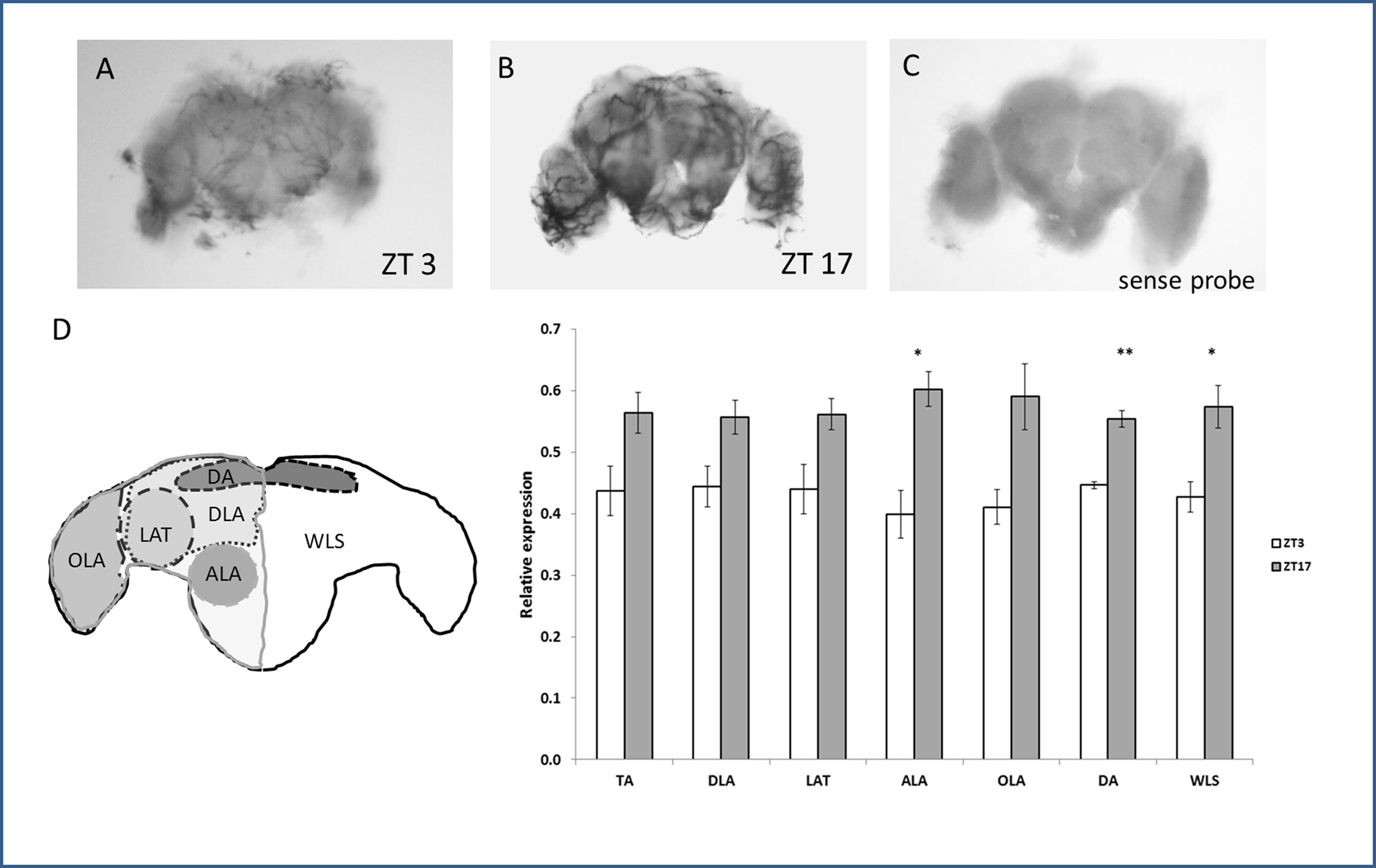

Supplement: Figure S1 — Whole mount in situ hybridization in Ae. aegypti male brains using a period RNA probe. Brains collected at ZT3 and ZT17 corresponding respectively to trough (A) and peak (B) per gene expression in LD12∶12 according to previous qPCR data [14], were hybridized with a digoxigenin-labeled RNA probe. It is worth to note the labeling pattern seen with the per probe is in antiphase with the labeling pattern seen in Figure 1. Sense probe (C) was used as control. To compare the differences between peak (ZT17) and trough (ZT3) period expression in brains, we arbitrarily divided the brain in regions (left) and analyzed them using ImageJ software. A t-test was used to compare the two time points for the different regions and some regions presented significant differences between the two time points, as the Antennal lobe area (P<0.05) and the Dorsal area (P<0.01), marked by asterisks (right). The other regions presented a borderline statistical difference. TA: total area, DLA: Dorsolateral area; LAT: Lateral area; ALA: Antennal lobe area; OLA: Optical lobe area; DA: Dorsal area; WLS: Whole Left side. Brain scheme was based on Ignell and coauthors work [44]. The period probe synthesis was done in the same way as the cycle one (See Materials and Methods), but using the following primers: Forward –5′ GAGCTCCATATTTTGAGACATC 3′ and Reverse: 5′ TATGAAAGACCGTCCAAGC 3′. (TIF) [file pone.0052559.s001.tif]
